# New insights on the interaction between m^6^A modification and non-coding RNA in cervical squamous cell carcinoma

**DOI:** 10.1186/s12957-023-02907-z

**Published:** 2023-01-30

**Authors:** Guqun Shen, Fen Li, Yan Wang, Yongmei Huang, Gulibiya Aizezi, Jinrui Yuan, Cailing Ma, Chen Lin

**Affiliations:** 1 The Second Department of Gynecology, Affiliated Tumor Hospital of Xinjiang Medical University, Urumqi, 830011 China; 2 Xinjiang Key Laboratory of Oncology, Affiliated Tumor Hospital of Xinjiang Medical University, Urumqi, 830011 China; 3 Operating Theatre, Affiliated Tumor Hospital of Xinjiang Medical University, Urumqi, 830011 China; 4 State Key Laboratory of Pathogenesis, Prevention, and Treatment of High Incidence Diseases in Central Asia/Department of Gynecology, The First Affiliated Hospital of Xinjiang Medical University, Urumqi, 830054 China; 5 Department of Pathology, School of Basic Medicine, Xinjiang Medical University, 789 Suzhou East Street, Urumqi, 830011 China

**Keywords:** CSCC, m^6^A, METTL3, lncRNA METTL4-2, YTHDF1

## Abstract

**Background:**

*N*^6^-Methyladenosine (m^6^A) and long non-coding RNAs (lncRNAs) are both crucial regulators in human cancer growth and metastasis. However, their regulation on cervical squamous cell carcinoma (CSCC) is largely unclear. The present study aimed to explore the role of m^6^A-associated lncRNAs in CSCC.

**Methods:**

We screened the expression of methylation modification-related enzymes in CECC samples from TCGA. The qRT-PCR was used to detect METTL3 and lncRNA METTL4-2 expression. The biological activities of METTL3 in CSCC cells were evaluated by CCK-8, colony formation, transwell, wound healing, and xenograft tumor assays, respectively. The SRAMP tool was used to screen m^6^A modification sites of METTL4-2. Finally, the quantitative analysis of m^6^A modification was carried out by MeRIP.

**Results:**

METTL3 expression was upregulated in CSCC cells and tissues. Biological function and function loss analysis indicated that METTL3 promoted the migration and proliferation of CSCC cells. In addition, METTL3 promoted CSCC tumor growth in vivo. Mechanically, METTL3 installed the m^6^A modification and enhanced METTL4-2 transcript stability to increase its expression. Meanwhile, the m^6^A “reader” YTHDF1 recognized METTL4-2 installed by METTL3 and facilitated the translation of METTL4-2.

**Conclusions:**

In conclusion, our study highlights the function and mechanism of METTL3-induced METTL4-2 in CSCC. These findings support that METTL3-stabilized METTL4-2 promoted CSCC progression via a m^6^A-dependent modality, which provides new insights into therapeutic strategies for CSCC.

**Supplementary Information:**

The online version contains supplementary material available at 10.1186/s12957-023-02907-z.

## Introduction

Cervical cancer (CC) is a human cancer with a high incidence rate of women, especially in developing countries [1]. In the report in 2018, it has been pointed out that CC accounts for about 3.2% of all new cancer cases and 3.3% of cancer deaths [2]. More importantly, the proportion of cervical squamous cell carcinoma (CSCC) has reached 90% of CC cases [3]. Studies have found a strong link between CC and human papillomavirus (HPV) infection [4]. Although the increase of HPV vaccination rate and the popularization of the HPV screening system have gradually accelerated the early diagnosis of CC and clarified the basis for diagnosis, the HPV infection rate is very high worldwide [5, 6]. Therefore, it is necessary to elucidate the molecular mechanism of CSCC occurrence and development, which may contribute to better study the therapeutic targets of CSCC.

Long non-coding RNAs (lncRNAs) refer to a special type of RNAs, with a length of 200 nt that do not encode proteins or have a limited protein-coding capacity [7]. Abnormal expression of lncRNAs has proposed to multiple biological functions, such as the initiation, metastasis, and epithelial-mesenchymal transformation (EMT) [8, 9]. In recent years, multiple studies have exhibited that lncRNAs are actually key determinants or diagnostic criteria of human cancer [10, 11]. Especially in CSCC, lncRNAs can also alter various malignant phenotypes [12, 13]. For example, the overexpressed lncRNA BLACAT1 in CSCC promoted cell invasion, proliferation, and migration to accelerate cancer progression [12, 14]. Therefore, lncRNA-mediated key regulation may greatly affect the progress of CSCC.

*N*^6^-Methyladenosine (m^6^A) is an epigenetic chemical modification existing in a variety of RNA molecules, which is the most abundant in eukaryotes [15]. In addition, m^6^A is a dynamic and reversible mode of RNA modification mainly mediated by RNA methyltransferases (m^6^A writers), RNA demethyltransferases (m^6^A erasers), and RNA methylation modification binding proteins (m^6^A readers) [16, 17]. METTL3 is a key catalyst for m^6^A modification [18], which display carcinogenic effects in breast cancer [19], endometrial cancer [20], and gastric cancer [21]. After different “readers” recognize gene m^6^A modification sites, cells will trigger different types of RNA metabolic effects, including mRNA structure, maturation, splicing, export, translation, and stability [22, 23]. YTH domain family 1 (YTHDF1) is the most functional “m^6^A reader” currently known, which can specifically recognize m^6^A-modified mRNAs and recruit multiple RNA-binding proteins to process mRNAs to improve translation efficiency [24]. For instance, the m^6^A reader YTHDF1 recognized the m^6^A residue on the CPCP1 3′‐UTR installed by METTL3 to facilitate CDCP1 translation in NSCLC [25]. In addition, growing evidence confirmed the regulation of m^6^A on EMT and metastasis of malignant tumors. For instance, m^6^A-related lncRNA RP11 triggered EMT of colorectal cancer through upregulation of Zeb1 [26]. However, the molecular mechanism of m^6^A on EMT and metastasis of CSCC has never been reported.

In our report, the role of METTL3 in the pathogenesis of CSCC was investigated. Data from this study confirmed that YTHDF1 recognized m^6^A-related lncRNA METTL4-2 by METTL3, improved the translation efficiency of METTL4-2 mRNA, and promoted its expression, thus promoting the EMT process of CSCC.

## Materials and methods

### Clinical tissues

Two group clinical patient samples: (1) 8 CSCC tissues and 8 corresponding normal tissues were collected from May 2019 and June 2020; (2) 8 CSCC metastatic tissue samples and 8 non-metastatic tissues were collected from October 2019 to May 2021. None of the patients had undergone preoperative chemoradiotherapy. The study protocol was approved by the Affiliated Tumor Hospital of Xinjiang Medical University. The written consent had obtained from each participant before the study.

### Cell culture

The human CSCC cell lines (SiHa and C33A) and human normal cervical epithelial cell line (H8) were obtained from the Chinese Academy of Sciences Cell Bank (Shanghai, China) and American Type Culture Collection (Manassas, VA, USA). The cells were cultured in DMEM (Gibco, USA) containing 10% fetal bovine serum (Gibco, USA), 100 U/ml penicillin, and 100 mg/ml streptomycin. These cells were incubated in a 5% CO_2_ incubator at 37°C.

### Cell transfection

All cell transfections were performed by Lipofectamine 2000 reagent (Invitrogen, USA), including Sh-NC, Sh-METTL3#1, Sh-METTL3#2; Vector-OE, METTL3-OE; lncRNA METTL4-2-OE; Sh-NC, Sh-YTHDF1#1; and Sh-YTHDF1#2; (GenePharma, Shanghai, China). Cells were harvested at 48 h post-transfection for the following experiments.

### Animal experiment

Thirty-six BALB/c nude mice, 5-week-old, weighing about 18–20g, were fed on a 12-h light/dark schedule with free food and water. In order to conduct in vivo experiments, the lentivirus was customized by Shanghai Gene Pharma, including Sh-NC, Sh-METTL3#1, NC-OE, METTL3-OE, and lncRNA METTL4-2-OE. Subsequently, we subcutaneously injected C33A cells with adjusted gene expression into the axilla of mice. When a tumor mass appears (1 week), the tumor volume was measured with a vernier caliper every other week. Use the following formula to calculate: tumor volume (V) = (length × width^2^)/2. After 28 days, the mice were sacrificed and the tumors were isolated, measured the size and weight, and analyzed by a digital camera.

### qRT-PCR

We use TRIZOL reagent (Thermo Fisher, USA) to extract total RNA from cells and tissues. For cDNA, reverse transcription was performed using the PrimeScript RT reagent Kit (TaKaRa, Otsu, Shiga, Japan). Gene expression was detected using a fluorescence quantitative PCR instrument (Analytick Jena A G, Germany). The reaction was conducted under the guidance of a fluorescent quantitative RT-PCR kit (SYBR Green, Bio-RAD, USA). The reaction conditions were described as 30 s denaturation at 94℃, followed by 35 cycles of denaturation at 95°C for 10 s, annealing at 58°C for 20 s, and extension at 72°C for 1 min. U6 and GAPDH were used as the internal control. The primer sequences were METTL3-F: 5′-CTATCTCCTGGCACTCGCAAGA-3′; METTL3-R: 5′-GCTTGAACCGTGCAACCACATC-3′; METTL4-2-F: 5′-TCCTAATAAGCCATTCCAGTCATT-3′; METTL4-2-R: 5′-TCTGCTCCTTCCTGCTATCT-3′; YTHDF1-F: 5′-GCACACAACCTCCATCTTCG-3′; YTHDF1-R: 5′-AACTGGTTCGCCCTCATTGT-3′; U6-F: 5′-CTCGCTTCGGCAGCACATATAC-3′; U6-R: 5′-AACGCTTCACGAATTTGCGTGTC-3′; GAPDH-F: 5′-GTCTCCTCTGACTTCAACAGCG-3′; and GAPDH-R: 5′-ACCACCCTGTTGCTGTAGCCAA-3′. All data were analyzed by adopting the 2^−ΔΔCT^ method. ΔCT (test) = CT (target, test) − CT (internal control, test); ΔCT (calibrator) = CT (target, calibrator) − CT (internal control, calibrator); ΔΔCT = ΔCT (test) − ΔCT (calibrator).

### Western blot

The cells were lysed using RIPA lysis buffer (Synthgene, Nanjing, China) and then centrifuged × 12,000 g for 20 min at 4°C. The concentration of protein in the supernatant was examined by the BCA protein Kit (Beyotime Biotechnology, China). Approximately 50 μg of protein was firstly placed on 10% SDS-PAGE and then shifted to a PVDF membrane. The proteins were incubated with the following primary antibodies at 4°C for 16 h: METTL3 (1:2000, Abcam, USA), E-cadherin, vimentin, N-cadherin, FN1 (1:1500, SBI, USA), and GAPDH (1:2000, Abcam, USA). GAPDH acts as a loading control. The membranes were washed with PBS for 3 times, and then corresponding secondary antibodies were added, followed by incubation for 1 h. Finally, the protein bands were quantified via ImageJ software (1.47V, NIH, USA).

### Cell viability assay

Cell counting kit-8 assay (Beyotime, Shanghai, China) was used to evaluate the effect of transfection on the proliferation of CSCC cells. The cells were seeded in 96-well plates at a density of 2000 cells/well and then CCK8 reagent (10 μL) was added to each well. After incubation for 3 h, the absorbance was measured at 450-nm wavelength with an automatic microplate reader (MolecμLar Devices, Shanghai, China).

### Colony formation assay

Briefly, C33A cells were grown in 6-well plates. Then, the cells (10^4^ per plate) were seeded on plates and allowed to grow for 10–14 days. Then, the cells were fixed with methanol for 15 min and stained by 0.2% crystal violet. The colony number was manually counted and photographed.

### Transwell assay

A transwell chamber (24-well chamber with an 8-µm pore) was used to detect the migration ability of C33A cells. The cells (1×10^4^ cells/well) were seeded in the upper chamber with a serum-free medium. Subsequently, we added a medium containing 10% FBS to the lower chamber. After being incubated overnight, we fixed the cells with methanol and stained using crystal violet. Finally, cells were observed under an inverted microscope.

### Wound healing assay

C33a cells were inoculated into 6-well culture plates and grew to confluence. We scraped the monolayer cells between two parallel straight lines in the middle of the culture plate with the tip of a sterile micropipette (0.5 mm) and washed culture plates with PBS to remove the floating cells. At 0 and 24 h after the scratch, the cell migration images were taken with an inverted microscope under the condition of 100 times magnification, respectively.

### Immunohistochemistry (IHC)

The expression level of METTL3 in CSCC tissues was detected by immunohistochemistry. Briefly, the sections (5μm) were hydrated with conventional dewaxing and then rehydrated with alcohol. They were then incubated using primary antibody anti-METTL3 (1:100, Abcam, UK) overnight at 4°C and then incubated using a biotin-labeled secondary antibody (1:200, Abcam, UK) at 37°C for 30 min. Subsequently, the sections were counterstained using hematoxylin. Finally, observe and take pictures under a microscope.

### HE staining

After mice were euthanized, CSCC tissues were collected and fixed. The sections (5μm) were stained using hematoxylin and eosin and dehydrated again (Olympus, Japan). Finally, histopathological characteristics were assessed under the microscope (CX31, Olympus, Japan).

### m6A RNA methylation quantification

The total RNA m^6^A level of C33A cells was detected using the EpoQuik^TM^ m^6^A RNA Methylation Quantification Kit (Colorimetric, No.#P-9005, Epigentek). In brief, the negative and diluted positive controls were then separately added to the assay wells with 200 ng of RNA and incubated for 90 min. The absorbance was monitored at 450-nm wavelength and the relative content of m^6^A was calculated by using the standard curve.

### MeRIP-qRT-PCR

For quantification of m^6^A-modified METTL4-2 levels, methylated RNA immunoprecipitation was performed as previously reported [25]. In brief, total RNA was extracted from C33A cells by TRIzol (Invitrogen, CA, USA). One microgram of m^6^A antibody (ab151230; Abcam) was coupled to the mixture of Protein A beads (Thermo Fisher Scientific, Waltham, MA). Later, m^6^A RNA immunoprecipitation was performed using a GenSeq™ m^6^A RNA IP Kit and was eluted for m^6^A MeRIP library construction. Subsequently, beads were washed twice with RIP buffer and then resuspended in lysis buffer. After rotation and elution, the RNA enrichment was extracted using the PureLink RNA extraction kit (Ambion, TX, USA).

### RNA stability

RNA stability was determined as previously described [27]. Briefly, cells were treated with 5 mg/ml actinomycin D (No.#A9415, Sigma) and then collected at different time points. RNA was then extracted and detected by qRT-PCR. The RNA levels at different times were calculated and normalized to GAPDH.

### Statistical analyses

Data are presented as mean ± standard deviation (SD) of three independent experiments. GraphPad Prism 7.0 was used for data analysis. When comparing the two groups, Student’s *t*-test was used for statistical analysis. Besides, one-way ANOVA or two-way ANOVA was conducted followed by post hoc Dunnett’s test to calculate the mean difference between more than two groups. The Wilcoxon rank sum test was used to compare gene expression between CSCC tumor samples and paratumor samples in the TCGA dataset. *P* < 0.05 was considered statistically significant.

## Results

### METTL3 is upregulated in CSCC tissues and cells

**Fig. 1:**
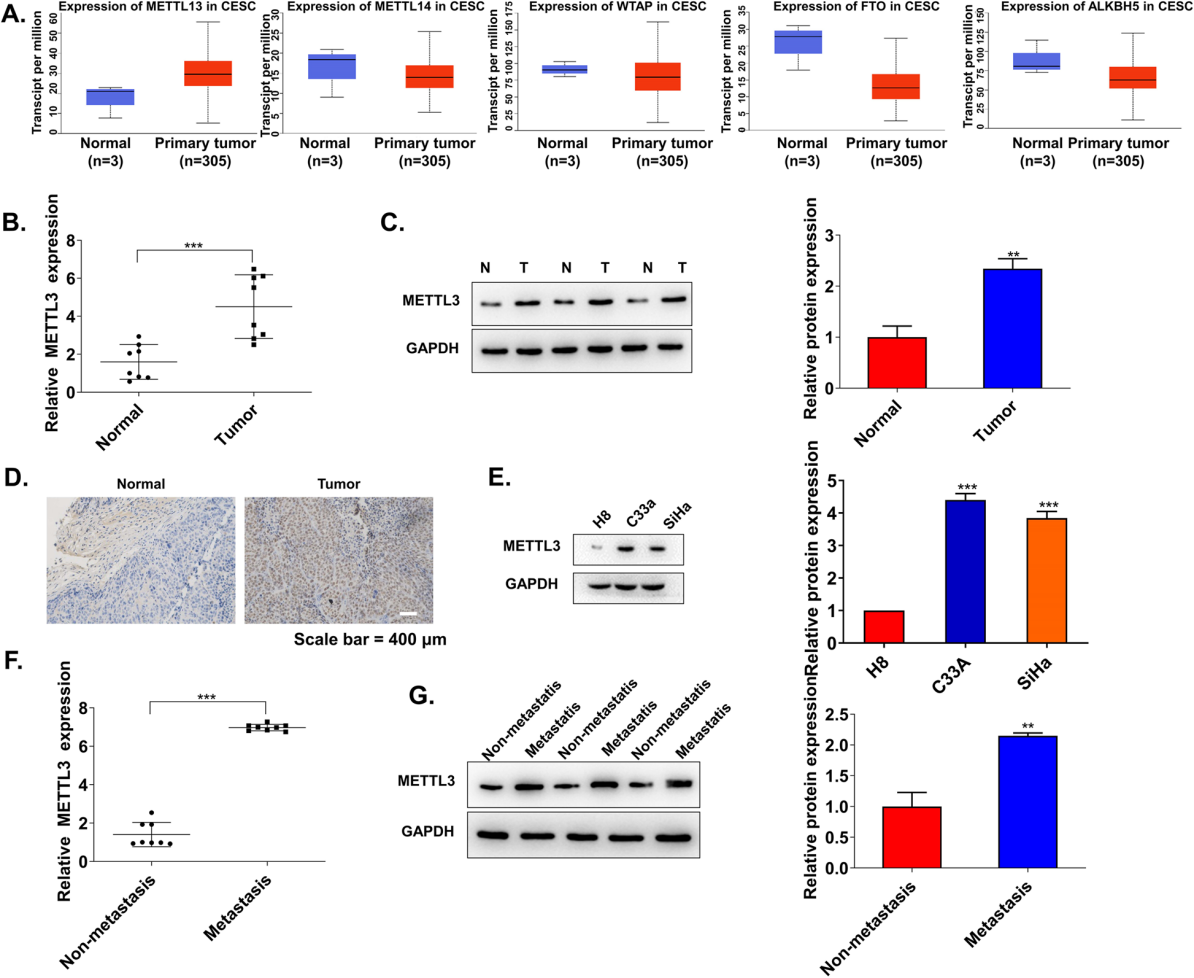
METTL3 is upregulated in CSCC tissues and cells. **A** Expression levels of m^6^A writers (METTL3, METTL14, and WTAP) and erasers (FTO and ALKBH5) were detected in the TCGA database. **B** qRT-PCR detected METTL3 expression in CSCC tissues and normal tissues (*n*=8). **C** The protein expression of METTL3 was detected by western blot in CSCC metastatic tissues (T) and non-metastatic tissues (N) (*n*=3). **D** Immunohistochemistry (IHC) staining was used to reveal METTL3 protein expression in CSCC tissues (*n*=3). **E** The mRNA level of METTL3 in CSCC cell lines (SiHa and C33A) and normal cervical epithelial cells (H8) was detected by qRT-PCR (*n*=3). **F** METTL3 expression in CSCC metastatic tissues and non-metastatic tissues was detected by qRT-PCR and **G** western blot (*n*=3). **p < 0.01; ***p < 0.001

Plenty of researches have confirmed that m^6^A is highly variable and associated with tumorigenesis [28]. m^6^A affects the outcome of gene expression, this process of which includes localization, transcription, translation, and final decay, and can be jointly regulated by “writers,” “erasers,” and “readers” [29–31]. Based on this, we first searched the expression of m^6^A writers (such as METTL3, METTL14, and WTAP) and erasers (such as FTO and ALKBH5) through the TCGA database. The results found that METTL3 was overexpressed in CSCC tumors (Fig. 1A). qRT-PCR, western blot, and IHC also proved the above results (Fig. 1B–D). Consistently, METTL3 expression was significantly higher in CSCC cell lines (SiHa and C33A) than that in normal cervical epithelial cell lines (H8), especially C33A (Fig. 1E). Subsequently, METTL3 expression in non-metastatic and metastatic tissues was detected by qRT-qPCR and western blot. METTL3 levels were obviously upregulated in metastatic tissues (Fig. 1F, G). These results suggested that METTL3 was overexpressed in CSCC tissues, especially in metastatic tissues.

### Abnormal expression of METTL3 affects the progression of CSCC

**Fig. 2:**
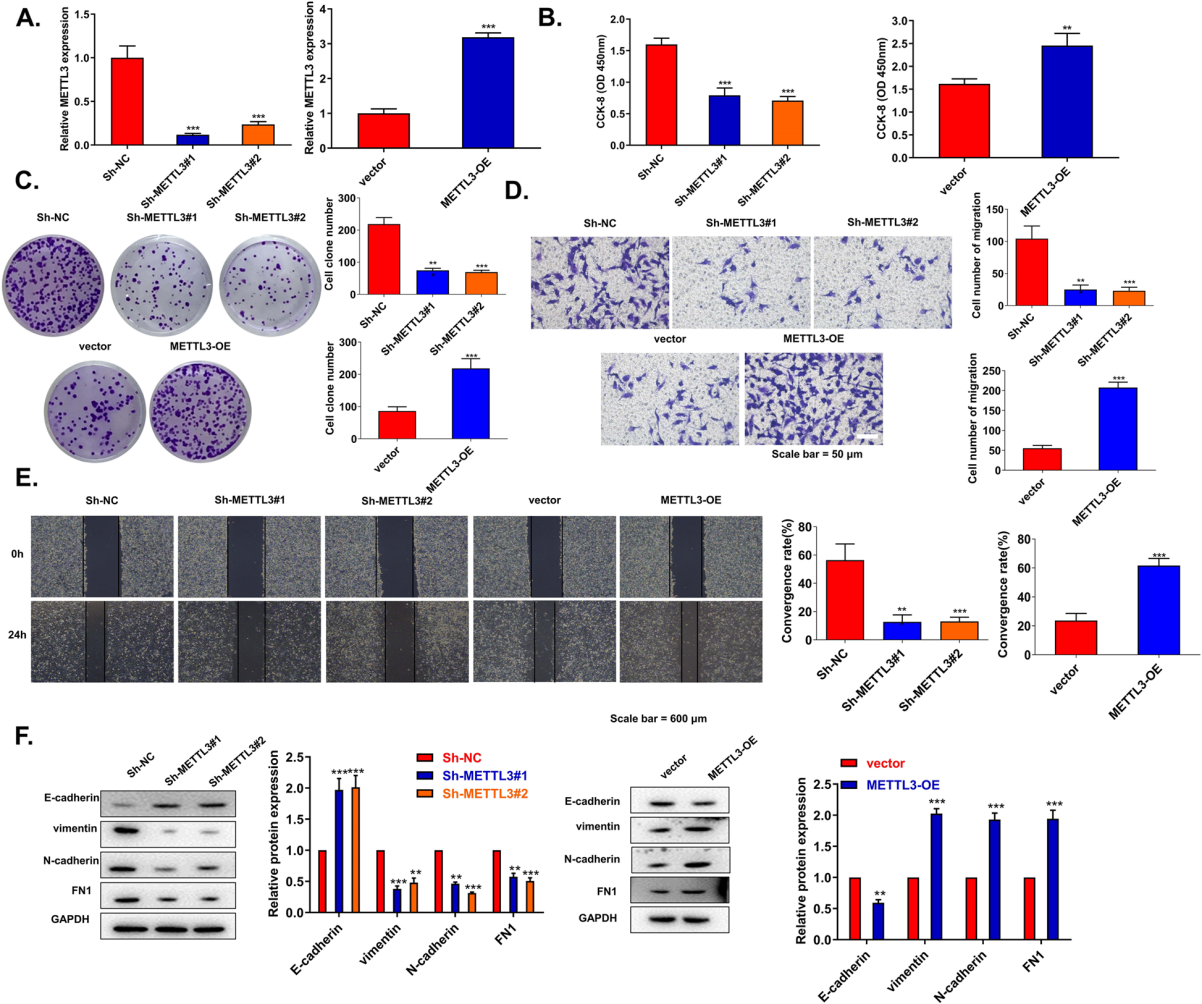
Abnormal expression of METTL3 affects the progression of CSCC. **A** METTL3 expression was examined using qRT-PCR in C33A cells transfected with sh-NC, Sh-METTL3#1, Sh-METTL3#2, and METTL3 OE (*n*=3). **B** The proliferation of C33A cells was detected with a CCK-8 kit (*n*=3). **C** Colony formation assays (*n*=3). Cell migration and invasion were detected by **D** transwell assay and **E** wound healing assay (*n*=3). **F** The levels of E-cadherin, N-cadherin, vimentin, and FN1 in C33A cells were detected by western blot (*n*=3). **p < 0.01; ***p < 0.001

To further reveal the potential effect of METTL3 on CSCC cells, we studied the biological functions of METTL3 in C33A cells. The knockdown and overexpression efficiency of METTL3 was measured by RT-qPCR. METTL3 expression was significantly reduced in Sh-METTL3#1/#2 transfected cells. However, METTL3 level was significantly increased in METTL3 OE transfected cells (Fig. 2A). Based on CCK8 and clone formation experiments, the knockdown of METTL3 significantly attenuated cell growth, whereas overexpression of METTL3 remarkably increased cell growth (Fig. 2B, C). In further transwell and wound healing assays, METTL3 knockdown significantly attenuated the migration and invasion ability of cells, whereas METTL3 overexpression significantly increased the migration and invasion ability (Fig. 2D, E). Furthermore, METTL3 deficiency induced downregulation of vimentin, N-cadherin, FN1, and upregulation of E-cadherin, while overexpression of METTL3 had the opposite effects (Fig. 2F). All these results elucidated that the abnormal expression of METTL3 in CSCC cells affects the progression of CSCC in vitro.

**Fig. 3:**
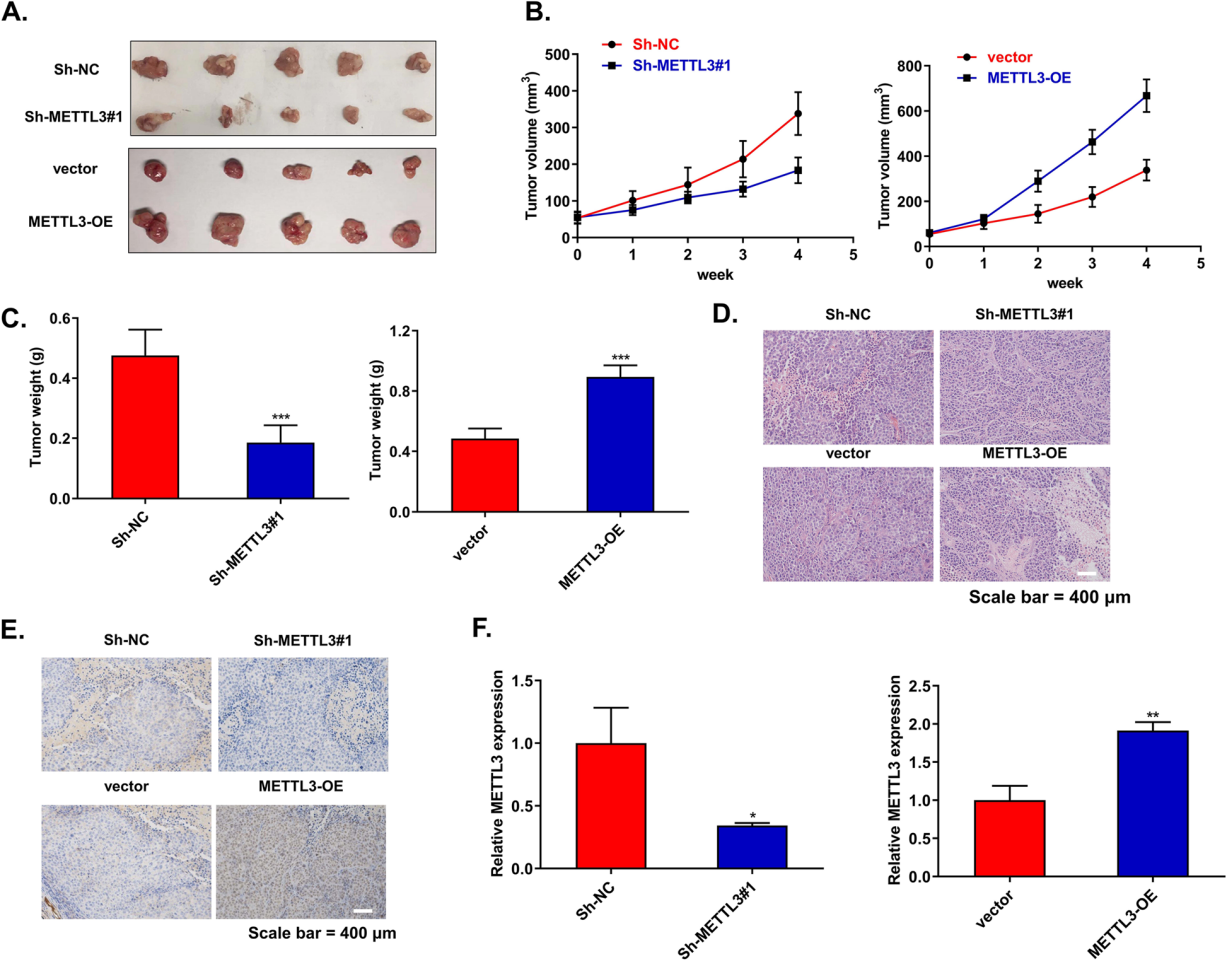
Abnormal expression of METTL3 affects the growth of CSCC tumors. **A** Typical CSCC tumors from mice after subcutaneous injection of C33A cells with overexpression or knockdown of METTL3 (*n*=5). **B**, **C** The volume and weight of CSCC subcutaneous tumors in mice after different treatments (*n*=5). **D** HE staining of tumor tissues (*n*=5). **E**, **F** METTL3 expression in CSCC tumor tissue was determined by IHC staining and qRT-PCR, respectively (*n*=5). *p<0.05, **p<0.01, ***p<0.001

Next, to further verify the effect of METTL3 on the CSCC progression in vivo, we firstly knocked down or overexpressed METTL3 in C33A cells. Subsequently, we subcutaneously injected these C33A cells into BALB/c nude mice. The data showed that compared with control xenografts, the knockdown of METTL3 slowed tumor growth, whereas the upregulation of METTL3 promoted tumor growth (Fig. 3A–C). The HE stained of CSCC tissue slides also revealed that compared with the controls, overexpression of METTL3 increased the malignant morphology of the denser tumor, while knockdown of METTL3 reduced the malignant morphology of the looser tumor (Fig. 3D). In addition, IHC staining and qRT-PCR showed that knockdown of METTL3 significantly reduced METTL3 protein and mRNA levels, whereas overexpression of METTL3 significantly increased METTL3 protein and mRNA levels (Fig. 3E, F). These results suggested that abnormal expression of METTL3 affects the progression of CSCC in vivo.

### METTL3 upregulates lncRNA METTL4-2 expression

**Fig. 4:**
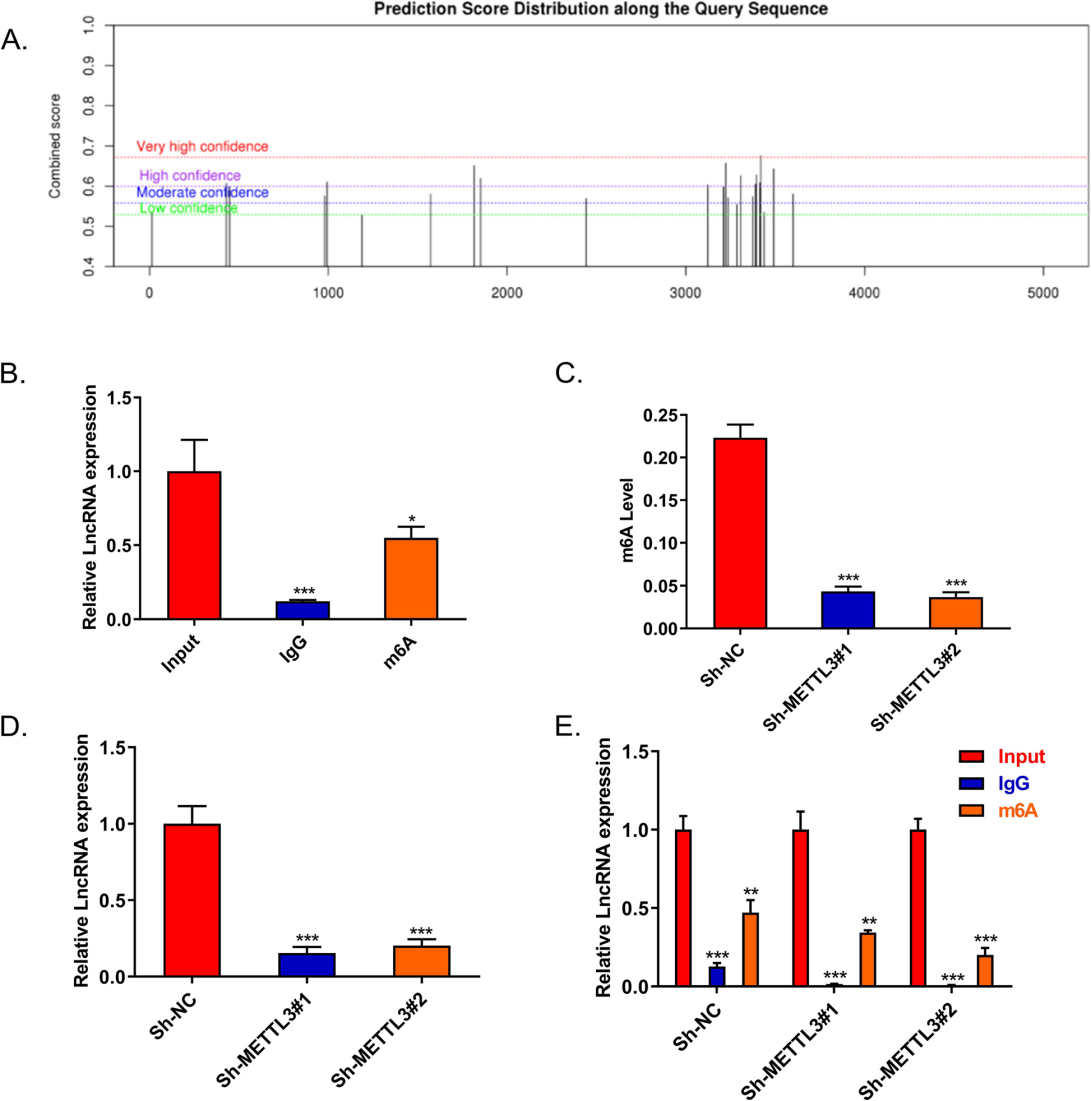
METTL3 enhances the stability of lncRNA METTL4-2 transcript and upregulates its expression. **A** The SRAMP tool was used to screen m^6^A modification sites of METTL4-2. **B** The enrichment of METTL4-2 followed by MeRIP-qRT-PCR assay in C33A cells (*n*=3). **C** m^6^A modification in C33A cells was conducted by colorimetric quantificational analysis (*n*=3). **D** The effect of METTL3 silencing on METTL4-2 expression was detected by qRT-PCR (*n*=3). **E** MeRIP‐qPCR demonstrated the m^6^A modification of METTL4-2 in C33A cells with METTL3 knockdown transfection (*n*=3). *p<0.05, **p<0.01, ***p<0.001

Recent advance in tumor epigenetic regulation has revealed the involvement of m^6^A in lncRNAs [26, 32]. We wanted to know whether m^6^A was associated with lncRNAs in CSCC. Therefore, in this study, we selected three CSCC tissues and matched normal tissues for lncRNA sequencing (Supplementary Table 1). We selected 6 upregulated lncRNAs with fold change over 200, among which MIR9-3HG [33] and KCNMB2-AS1 [34] were related to the development of CC, while the latter is stabilized by m^6^A modification to promote the growth of CC as a competitive endogenous RNA. In addition, the most differentially overexpressed lncRNA-NPHS2-6 has been studied in our previous work (data not published). Therefore, in this study, lncRNA-METTL4-2 that ranked second in fold change was selected to investigate the potential mechanism in CSCC. In the sequence-based RNA adenosine methylation site predictor (SRAMP) tool, we found that there were many m^6^A modification sites on METTL4-2 (Fig. 4A). MeRIP-qRT-PCR assay indicated that METTL4-2 was enriched in cells treated with anti-m^6^A antibody rather than IgG (Fig. 4B). Colorimetric quantificational analysis found that knockdown of METTL3 reduced the modification of m^6^A in CSCC cells (Fig. 4C). Next, whether METTL3 regulated METTL4-2 expression in CSCC cells was detected. It was found that METTL3 silencing significantly reduced the expression of METTL4-2 (Fig. 4D). In addition, MeRIP-qPCR demonstrated that the knockdown of METTL3 downregulated the m^6^A modification of METTL4-2 (Fig. 4E). In summary, these results suggested that METTL3 could upregulate METTL4-2 expression.

### YTHDF1 enhances the stability of METTL4-2 via m6A modification

**Fig. 5:**
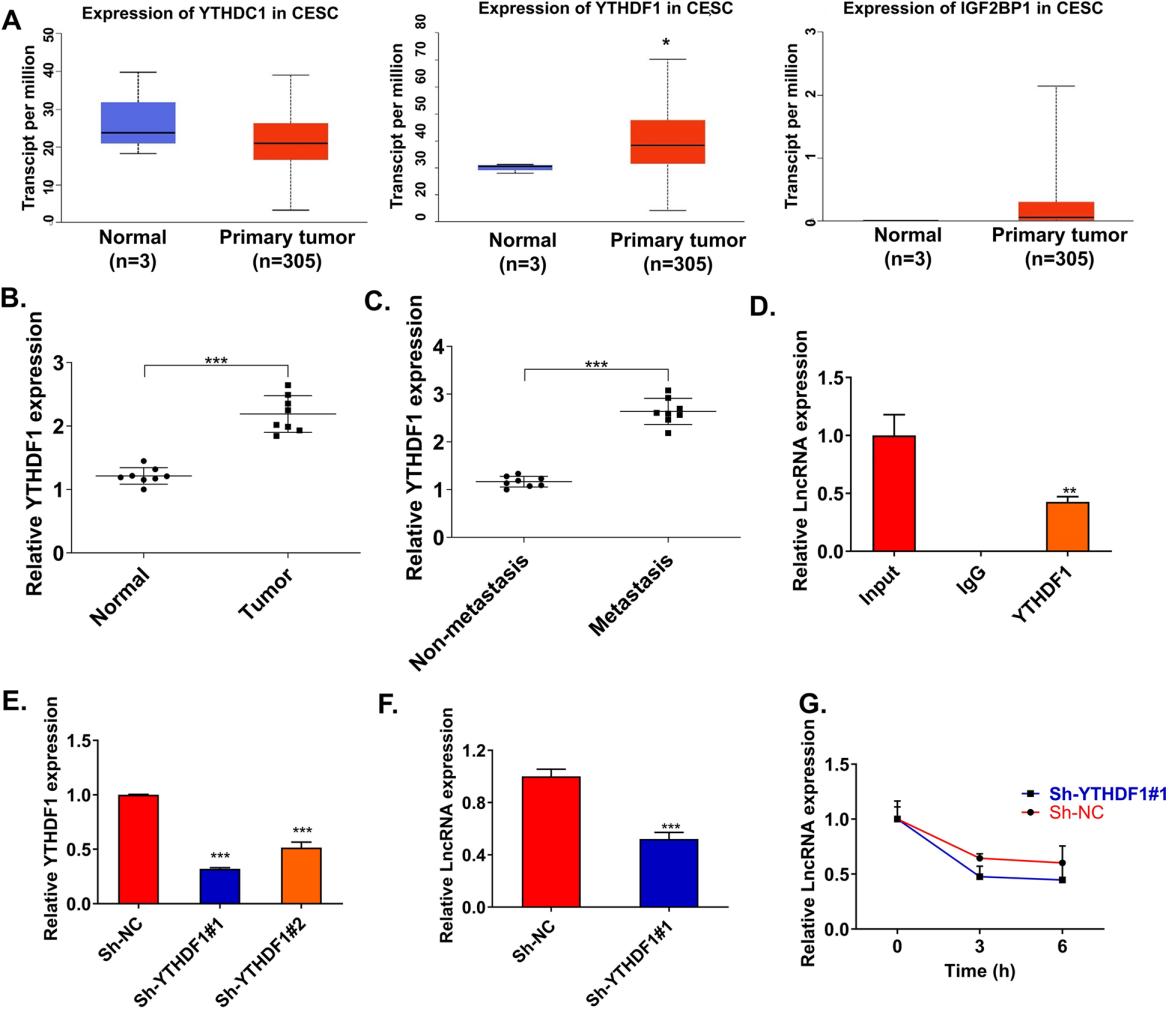
YTHDF1 enhanced the stability of METTL4-2 via m^6^A modification. **A** The expression of m^6^A “reader” (such as YTHDC1, YTHDF1, and IGF2BP) was detected by the TCGA database. **B** qRT-PCR detected YTHDF1 expression in CSCC tissues and normal tissues (*n*=8). **C** qRT-PCR detected YTHDF1 expression in CSCC metastatic tissues and non-metastatic tissues (*n*=8). **D** RIP assay was performed in CSCC cells using an anti-YTHDF1 antibody, followed by the qRT-PCR analysis of METTL4-2 enrichment (*n*=3). **E** The transfection efficiency of YTHDF1 silencing by qRT-PCR (*n*=3). **F** The effect of YTHDF1 silencing on METTL4-2 expression was detected by qRT-PCR (*n*=3). **G** RNA stability analysis of METTL4-2 in treated C33A cells (*n*=3). *p < 0.05; **p < 0.01; ***p < 0.001

Epigenetic m^6^A marks A have been reported to be recognized mainly by “readers” [29–31]. Therefore, we detected the expression of m^6^A “reader” (such as YTHDC1, YTHDF1, and IGF2BP) through the TCGA database. The result found that the YTHDF1 level was higher in the CSCC tumors than the normal tissues (Fig. 5A). Subsequent clinical samples showed that YTHDF1 expression was overexpressed in CSCC tissues, especially in the metastatic tissues (Fig. 5B, C). Then, we studied the effect of YTHDF1 on METTL4-2 in CSCC cells. MeRIP-qPCR assay revealed that METTL4-2 was abundantly enriched by the YTHDF1 antibody (Fig. 5D). Next, we downregulated the level of YTHDF1 and qRT-PCR was used to detect knockdown efficiency (Fig. 5E). RT-qPCR results showed that METTL4-2 expression was inhibited after YTHDF1 knockdown (Fig. 5F). RNA stability analysis found that YTHDF1 knockdown reduced the stability of METTL4-2 (Fig. 5G). These results clearly suggested that YTHDF1 enhanced the stability of METTL4-2 via m^6^A modification.

### METTL3 regulates METTL4-2 expression and promotes the progression of CSCC

**Fig. 6:**
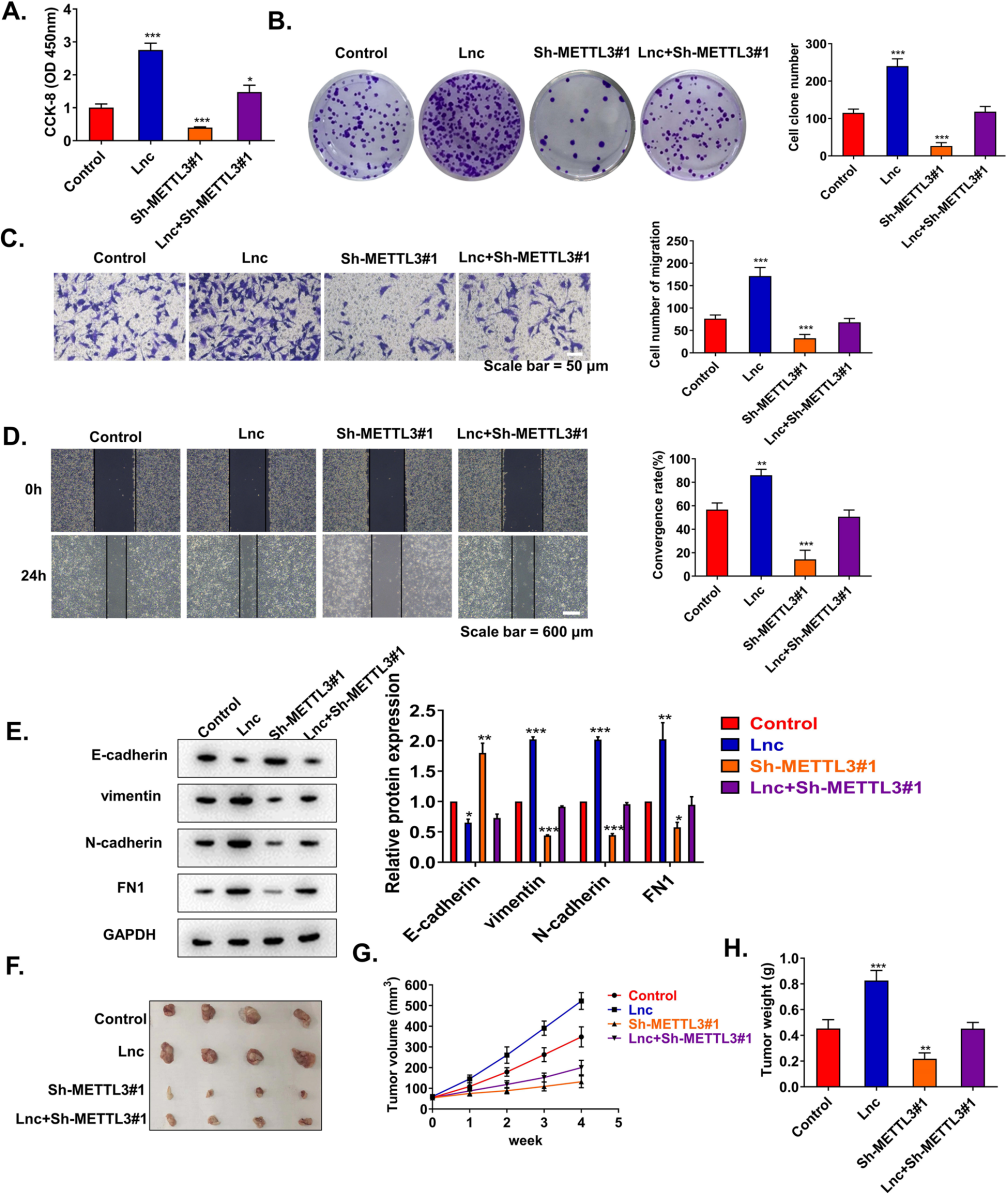
Functional complementarity experiments demonstrated that METTL3 regulates METTL4-2 expression in CSCC progression. We firstly constructed C33A cells transfected with Sh-NC, lncRNA METTL4-2 OE, Sh-METTL3#1, and lncRNA METTL4-2 OE+Sh-METTL3#1 treatment, respectively. In animal experiments, we inoculated C33A cells performed with the above treatments subcutaneously at the armpits of mice. **A** Cell viability of C33A cells was measured by CCK-8 assay (*n*=3). **B** Colony formation assays of C33A cells (*n*=3). **C** The invasion of C33A cells was assessed by transwell assay (*n*=3). **D** The migration of C33A cells of indicated treatment was assessed by wound healing assay (*n*=3). **E** The levels of E-cadherin, N-cadherin, vimentin, and FN1 in C33A cells were detected by western blot (*n*=3). **F** Typical CSCC tumors from mice after subcutaneous injection of C33A cells with different treatments (*n*=4). **G** Tumor volume was measured every week by growth curve (*n*=4). **H** Tumor weight was measured at the end of experiments (at the 5 weeks) (*n*=4). *p < 0.05; **p < 0.01; ***p < 0.001

Functional complementation experiments were performed to evaluate the regulatory effect of METTL3 on METTL4-2. We firstly constructed C33A cells transfected with Sh-NC, lncRNA METTL4-2 OE, Sh-METTL3#1, and lncRNA METTL4-2 OE+Sh-METTL3#1 treatment, respectively. The CCK8 assay, colony formation, transwell assay, and wound healing showed that upregulated METTL4-2 significantly increased the cell proliferation, migration, and invasion, while downregulation of METTL3 significantly reduced cell proliferation, migration, and invasion. Importantly, simultaneous knockdown of METTL3 while overexpression of METTL4-2 neutralized the above effects (Fig. 6A–D). Furthermore, overexpression of METTL4-2 induced downregulation of vimentin, N-cadherin, and FN1 and upregulation of E-cadherin, while knockdown of METTL3 induced upregulation of vimentin, N-cadherin, and FN1 and downregulation of E-cadherin. Consistently, during overexpression of METTL4-2, simultaneous knockdown of METTL3 neutralized the above effects to EMT-associated factors (Fig. 6E). METTL3 could regulate the expression of METTL4-2 and promote EMT in CSCC cells. To further confirm this conclusion in vivo, we subcutaneously injected C33A cells with adjusted gene expression into the axilla of mice. The results were consistent with the trend of tumor growth in vitro experiments (Fig. 6F–H). In summary, we confirmed that METTL3 could regulate METTL4-2 expression and promote the progression of CSCC in vivo and in vitro.

## Discussion

Growing evidence suggests that lncRNAs are closely related to the pathogenesis of CSCC [12, 35]. With the rapid development of epigenetics and molecular biology, m^6^A-related lncRNA regulation resulting in self-renewal of tumor cells has gradually attracted the attention of researchers [36, 37]. Here, we proposed an interesting highlight that m^6^A is involved in the pathogenesis of CSCC together with lncRNAs.

Nowadays, m^6^A has made significant progress in the regulation of various stages of the RNA life cycle. M^6^A methylase complex is encoded by several “writers” where METTL3 localized in the catalytic core [23]. Increasing evidence supports the concept that METTL3 serves as a special mediator in tumorigenesis. The dual effects of METTL3 are inhibiting or promoting m^6^A modification in tumors [38]. For instance, Li et al. found that the deletion of METTL3 might promote the proliferation, migration, and invasion of renal small cell carcinoma [39]. However, Lin et al. observed that the knockdown of METTL3 dramatically inhibited liver cancer cell growth and EMT [40]. The above evidence suggested that METTL3 may be a novel marker for the tumorigenesis, development, and survival of cancer. Interestingly, METTL3 expression was upregulated in clinic CSCC tissues. Further cell studies confirmed that METTL3 overexpression in CSCC cells promoted cell proliferation, migration, and invasion, while deletion of METTL3 resulted in the opposite results. Overexpression of METTL3 could effectively promote subcutaneous tumor growth in nude mice, while knockdown of METTL3 could inhibit subcutaneous tumor growth. In addition, a series of studies have presented that METTL3 is associated with the occurrence and lung metastasis of liver cancer [41], colorectal cancer [27], gastric cancer [42], bladder cancer [43], and glioblastoma [44]. In this study, our clinical study also found METTL3 was upregulated in metastatic CSCC tissues. These data indicated that METTL3 could drive CSCC tumorigenesis and neoplasm metastasis.

It is well known that METTL3-mediated m^6^A affects targeted mRNA or miRNA, involved in EMT and metastasis of cancer [42]. For example, METTL3 preferentially recognized the m^6^A residues of CPCP1 and promoted its translation [25]. However, there are few studies on m^6^A-modified lncRNAs in the cancer field. In NSCLC, METTL3-correlated m^6^A modification enhanced the stability of lncRNA ABHD11-AS1 transcript to upregulate its expression [45]. Consistent with the above conclusions, our study first selected lncRNA METTL4-2 as the research object through lncRNA sequencing. Moreover, for the regulation of lncRNA METTL4-2, we found that METTL3 could preferentially install the m^6^A-modified site of METTL4-2 and enhance its transcript stability, thereby upregulating METTL4-2 expression. This finding is an interesting highlight that the METTL3-m^6^A-METTL4-2 axis could modulate the tumorigenesis and metastasis of CSCC.

RNA methyltransferase determines the abundance of m^6^A methylation modification after gene transcription, while the ultimate direction of m^6^A-modified abnormal mRNA in cells is determined by RNA methylation-modified binding protein. YTHDF1 is the most effective “m^6^A reader” known at present, which can specifically recognize m^6^A-modified mRNA and recruit multiple RNA-binding proteins to process the mRNA to improve translation efficiency and stability [24]. For instance, YTHDF1 recognized and bound to YY1 and MDM2 through m^6^A modification, which further promoted YY1 and MDM2 translation, thereby inhibiting p53 activation [46]. However, the regulatory role of YTHDF1 in tumor-related lncRNA translation is rarely studied. After our careful research, YTHDF1 was significantly upregulated in CSCC tumors based on the TCGA database. Clinical samples also showed that YTHDF1 expression was significantly increased in CSCC tissues, especially in the metastatic tissues. Further RIP assay revealed that YTHDF1 promoted the methylation modification of METTL4-2. RNA stability analysis found that YTHDF1 knockdown reduced the stability of METTL4-2. These results clearly suggested that YTHDF1 enhanced the stability of METTL4-2 via m^6^A modification.

## Conclusion

Our study firstly demonstrated that YTHDF1 recognized METTL3-mediated m^6^A methylation of lncRNA METTL4-2 and improved the translation efficiency of METTL4-2, which thus promoted the EMT process of CSCC cells. In total, we investigated the functional roles and molecular mechanisms of METTL3 in CSCC. Besides, by highlighting the crucial role of METTL3, our discovery may pave the way for developing new therapeutic strategies against CSCC.

## Supplementary Information

**Additional file 1: Supplementary Table 1.** The top 6 lncRNAs from lncRNAs sequencing of CSCC tissues and matched normal tissues.

## Data Availability

The datasets used and/or analyzed during the current study are available from the corresponding author on reasonable request.
