# Supplementary material for: New insights on the interaction between m6A modification and non-coding RNA in cervical squamous cell carcinoma
Source: World J Surg Oncol. 2023 Jan 30;21:25. doi: 10.1186/s12957-023-02907-z (PMC9885588; doi:10.1186/s12957-023-02907-z)
Supplement: Supplementary file 1 — Additional file 1: Supplementary Table 1. The top 6 lncRNAs from lncRNAs sequencing of CSCC tissues and matched normal tissues. [file 12957_2023_2907_MOESM1_ESM.docx]

**Supplementary Table 1. The top 6 lncRNAs from lncRNAs sequencing of CSCC tissues and matched normal tissues.**

| lncRNA | Fold change | P value |
| --- | --- | --- |
| lnc-NPHS2-6:1 | 1503.877447 | 1.57E-05 |
| lnc-METTL4-2:6 | 736.4049752 | 0.000994436 |
| lnc-EEF2KMT-11:1 | 669.4188942 | 0.037862205 |
| MIR9-3HG:22 | 650.2966654 | 6.01E-08 |
| lnc-SPATA21-2:17 | 389.2298135 | 9.33E-07 |
| KCNMB2-AS1:3 | 204.7845096 | 0.015729651 |
